# Discrepancies in assessing home care workers’ working conditions in a Norwegian home care service: differing views of stakeholders at three organizational levels

**DOI:** 10.1186/s12913-015-0945-6

**Published:** 2015-07-25

**Authors:** Gunn Robstad Andersen, Rolf H. Westgaard

**Affiliations:** Department of Industrial Economics and Technology Management, Norwegian University of Science and Technology, Alfred Getz veg 3, SB1, 12.etg, N-7491 Trondheim, Norway

**Keywords:** Organizational discrepancy, Health care services, Home care services, Work environment, Organizational change, Interventions, Rationalization

## Abstract

**Background:**

The present study is a follow-up study of factors contributing to an undesirable quality of work environment and sick leave rate in the home care services in a Norwegian municipality. The underlying assumption is that organizational discrepancies in the perceptions and appraisals of significant factors and processes in an organization have detrimental effects on the management of the organization and on work environment conditions. Thus, the study aim is to explore potential organizational discrepancies in the appraisals of factors relating to home care workers’ working conditions.

**Methods:**

The study, using a mixed-methods design, comprised six home care units. It included survey responses of home care workers (80 respondents, response rate 54 %) and qualitative descriptions of stakeholders’ appraisals of organizational issues gathered through semi-structured interviews (33 interviews with stakeholders at three organizational levels).

**Results:**

Employees at different organizational levels in the home care services expressed divergent appraisals of factors related to the working conditions of home care workers, including impact of organizational measures (i.e. time pressure, work tasks, a new work program, organizational changes, budget model, budget allocation and coping strategies). Survey responses supported interview descriptions by home care workers. Results suggest that organizational discrepancy serve as an important barrier to a sustainable, well-functioning organization in general and to quality-enhancing changes to work procedures in particular.

**Conclusions:**

It is recommended to improve communication channels and facilitate the exchange of information across levels to ensure a common understanding of matters significant to the organization of the home care services and to the work environment of home care workers. The prevalence and impact of organizational discrepancy should be included in organization research, particularly when exploring explanatory factors of an unhealthy organization.

## Background

Research indicates that home care workers (HCWs) are at risk of a multitude of occupational stressors, health complaints and sick leave [1–9]. Interventions primarily aimed at reducing occupational exposures and improving health rarely succeed in reaching their stated objectives [10–13]. In 2003 the municipality in this study received orders from the Norwegian Labor Inspectorate due to a high level of unhealthy time pressure and high sick leave in the home care services (HCS). The municipality responded promptly by giving work environment interventions and other alleviating actions high priority. Findings, based on data from 2009, indicated that the work environment interventions in general were perceived by the HCWs to have a positive effect by improving targeted areas identified to cause work strain. However, concurrent changes induced through production system rationalization, such as unit mergers, restructuring, changes to middle- and executive management, and introduction of new technology and new work programs, resulted in negative exposure effects that negated the positive intervention effects, causing an overall deteriorated work situation [11]. Production system rationalization involves a continuous effort to reduce costs and improve quality of an organization’s output (e.g. services offered to patients), and has a predominant negative impact on worker health [10]. The majority of HCWs in the present case reported an increase in perceived time pressure from 2004 to 2009. They reported exposure to several occupational risk factors and a high prevalence of musculoskeletal health complaints [6]. Sick leave rate remained high the next two years; 19.1 % in 2011 vs. 18.1 % in 2009, compared to 15.6 % in 2004. Consequently, the HCS seems to be struggling with persistent challenges that are difficult to overcome.

As prolonged high sick leave represents high costs as well as human suffering, it is a puzzle that HCS administrators have not been able to deal with the problem in the aftermath of the unsuccessful initial interventions. A possible explanatory factor is that decision-makers at municipal level have difficulties in recognizing or accepting the working conditions of workers at frontline. Organizational distance, insufficient inter-level exchange of information, and lack of insight in the interruptive influences of rationalizations have been suggested as key factors in this regard [11]. Stakeholders at different organizational levels may have divergent and potential conflicting perceptions and appraisals of organizational quality and productivity measures (i.e. *organizational discrepancy*); or holding incompatible “mental models” on organizational matters [14]. Literature on the prevalence and impact of organizational discrepancy is rather scarce, but existing research points to discrepancies in perceptions across organizational levels as potentially causing several detrimental consequences [15–18], such as obstructing effective implementation and leading to diversity in intervention experiences and outcomes [14].

The present study aims to explore potential discrepancies at three levels of an organization in assessing factors relating to HCWs’ working conditions. The underlying assumption is that organizational discrepancies in the perceptions and appraisals of significant factors and processes in the organization have detrimental effects on the management of the HCS and work environment conditions of HCWs. To explore this, interviews with stakeholders at all organizational levels (municipal representatives, home care unit leaders and HCWs) and survey responses of HCWs are included in the present study. The introduction of a new work program in the period 2009–2011 was of special interest in the survey, as findings presented in a previous publication indicated that work programs could have a significant impact on the HCWs’ workday [11], and informal conversations indicated inter-level discrepancies in this regard.

## Method

The study has a mixed-method design by using a combination of quantitative estimations (of perceived change in time pressure the last 2 years and perceived impact of the new work program introduced to the HCWs), and qualitative descriptions of the work situation in the HCS perceived by stakeholders at three organizational levels (municipal representatives, home care unit leaders and HCWs).

### Case description

In Norway, the municipalities are responsible for social welfare and health care of residents. The present HCS is organized by a two-level system. The municipal level constitutes the superior ‘administrative’ authority responsible for ensuring overall good quality of the services. The home care units are separated geographically, and each unit leader is responsible for managing the unit budget and organizing the daily operations. The HCWs are the frontline workers performing the services.

There has been a marked increase in home care services given to patients of all ages with a multiplicity of diagnoses and disabilities. An individual assessment executed by the municipal health and welfare office determines what medical help patients are entitled to receive. Each patient is given an ADL score (“Activities of Daily Living”) based on functional level, which constitutes the basis for money and time allocation to the units. An individual resolution contains specified activities for the HCWs to carry out in the patient’s home (labeled ‘direct time’ activities), such as putting on support stockings, giving medications, assisting with morning routines, measuring insulin level, wound treatment and so on. A specified amount of time is allocated to each task on the work list, typically varying from 10 to 45 min. Time estimation per assignment is decided at unit level and may be altered according to changes in patients’ functional level. However, the ‘norm’ from which time is allocated to the units is based on centrally determined ADL scores and the HCS’ total time production over a certain period of time.

A normal work day is a 7.5 h shift and may consist of 10 to 27 visits. Additionally, a considerable part of the workday involves transferring between patients and administrative tasks such as documentation and report writing (all labeled ‘indirect time’ activities), yet these activities are not explicitly specified in the work list. A percentage distribution of 70 (‘direct time’) - 30 (‘indirect time’) has generally been required to prevent deviation from unit budgets. A new work program introduced to the HCS in the period 2009–2011 has had consequences on the HCWs’ workday. The program involves specification and systematization of HCWs’ work duties and responsibilities at patients’ discharge from hospital as well as follow-up of patients’ overall functioning in their homes. The work tasks related to the program involve observations, assessments and documentation (i.e. indirect tasks), formalized in checklists that serve as tools for quality assurance through standardization of services.

### Participants

At study start in 2009, 6 (of 11) units signed up for participation (here entitled unit A-F). In the follow-up survey of 2011, one unit (unit E) was omitted due to low response rate. HCWs employed in a ≥ 50 % position were invited to fill in a questionnaire. Interview informants were selected through purposive sampling based on seniority (minimum 7 years) and employment fraction (≥50 %). Table 1 shows detailed information concerning data collection and participation during the study period. The present study is based on the follow-up questionnaire in 2011 and all interviews. The key informant is a HCW affiliated with a unit not part of the study. Through self-initiated time studies he gained comprehensive knowledge concerning actual time distribution to various work tasks during a typical work day, and was thereby considered to be an important informant in the present study.

**Table 1:** Data collection during the study period

	2009	2010	2011	2012
Questionnaire	(*n* = 138/181^a^)		*n* = 80/148^d^	
Interview	17 HCWs^b^	2 ML		5 UL^e^
	5 UL^c^	1 ED		1 ML
	1 ML	1 KI		

*HCWs* home care workers, *UL* unit leader, *ML* municipal level representatives, *ED* economy department, *KI* key informant

^a^Results presented in a previous publication [6]. Parenthesis indicating data not included in the present study

^b^Material also analyzed in a previous publication [11]. Data reanalyzed and included in the present study

^c^Units A–D, F

^d^54.05 % response rate

^e^Units A–C, E, F

### Procedure

Initial conversations were carried out with an inspector of the Norwegian Labor Inspectorate, three representatives of the municipality and all six unit leaders to gain insight in aspects of the HCS such as the organization of work duties, organization-specific work demands and significant changes and events relevant for the composition of interview guide and questionnaire. Prior to the data collection, one of the researchers participated on staff meetings at each unit to present the study and give practical information about participation. Questionnaires and informed consent forms were placed in individual envelopes and sent to the units where the unit leaders handed them out to the HCWs. The HCWs individually returned the filled-in questionnaire and informed consent form to one of the researchers by mail, and were remunerated with NOK 200 (24€). The data collection was carried out between May 31 2011 and December 8 2011, and was finally closed on February 22 2012. A written inquiry concerning interview participation was placed in selected personal shelves. Participating HCWs filled-in an informed consent form and were remunerated with NOK 300 (36€). The study was approved by the municipal executive, the Regional Committees for Medical and Health Research Ethics (REC) (no. 4.2009.19) and Norwegian Social Science Data Services (NSD) (no. 21036).

### Questionnaire

The questionnaire comprised altogether 52 items. The present study utilizes a self-formulated item regarding perceived changes in time pressure the last 2 years. Response categories ranged from 1 (considerable less) to 5 (considerable more) with a neutral mid-point, recoded to a three-point response scale (“less”, “no change” and “more”). The question “If change, why?” enabled open comments. Also, respondents were asked to evaluate the impact of the new work program on their work situation. Response categories ranged from 1 (no impact) to 5 (considerable impact) recoded to a three-point response scale (“minor impact”, “some impact” and “considerable impact”). The question “In what ways?” enabled open comments. IBM Statistical Package for Social Sciences (SPSS) version 19 was used to compute frequency distributions. The open comments were briefly formulated by the respondents, thus categorization and enumeration of responses was straight forward.

### Interviews

The interviews with HCWs and unit leaders lasted approximately 1 h, and slightly longer for municipal representatives. All interviews were carried out individually, audio-recorded and later transcribed. The interview guides covered topics concerning work environment of HCWs, work tasks, budget system, and perceived changes in such the last years. The paramount questions were “How do you perceive your work situation today?” and “How do you perceive changes in your work situation to affect you and your work?” Main questions were followed by probe questions such as “Can you give an example of this?”, “What do you think caused this?” and so on to stimulate rich descriptions. The interview guides for all levels covered the same topics in order to enable inter-level comparisons. Thus, unit leaders and municipal representatives were asked the same questions as the HCWs yet from their own perspective with regards to the HCWs’ work situation and the organization of the HCS. The interview with the representative from the economy department, however, was less focused on working conditions at frontline, but rather on the allocation model (with regards to time).

At two occasions after the interviews were carried out, a preliminary understanding of the topics in question, together with new questions where information was lacking, was emailed to unit leaders and municipal staff in order to clear-up possible misunderstanding (mainly involving financial and organization issues). Clarification was also requested when needed during the interviews with HCWs.

### Qualitative analysis

The interviews of HCWs from 2009 were reanalyzed and supplemented with interviews of unit leaders and municipal representatives with the purpose of exploring potential organizational discrepancies. Interview topics were similar across the different times of data collection. Descriptions presented by informants interviewed twice (i.e. unit leaders and municipal representatives) did not differ with regard to time of interviewing. The interview data was analyzed by Template analysis as this analyzing technique “works particularly well when the aim is to compare the perspectives of different groups of staff within a specific context” ([19], p.257). Hence, the data material was separated by organizational level during the analyzing process in order to explore potential inter-level similarities and discrepancies. The software QSR NVivo 9 (http://www.qsrinternational.com/products_nvivo.aspx) was utilized to aid in organizing and examining the data. The interview guide and findings presented in a previous study [11] served as basis for the initial template consisting of two higher-order themes: 1) “Strenuous work situation for HCWs” and 2) “Economy”; themes initially indicated to be characterized by inter-level discrepancies. The analyzing process was carried out by identifying higher-order themes and further scrutinizing the contents of these themes to identify and differentiate lower-order themes, resulting in a final template presented in the results section. This final template served as basis for interpretation and illumination of the data, in line with recommendations by King [19].

## Results

### Quantitative results

Table 2 shows survey responses on perceived changes in time pressure (A) and impact of the new work program (B). For time pressure, the left column shows the distribution of scale responses regarding changes in time pressure (1 missing). The majority of HCWs perceived increased time pressure. The categorization of open comments concerning *what caused* the perceived change in time pressure is presented in the right column, with the large majority of factors related to increased work demands (i.e., due to number and characteristics of patients).

**Table 2:** Summary of survey responses (*N* = 80). A. Perceived change in time pressure (left column) and factors causing change in time pressure (right column). B. Impact of new work program on work situation (summary labels in left column, explanatory items in right column). Number of responders (left column) and responses (right column) in parentheses

	Categorization of open comments
A. Time pressure^a^	
Increased^b^ (45)	Efficiency demands (14)
	Increased patient group/ heavier cases (12)
	Increased workload (10)
	New work program (4)
	No comments (8)
Decreased (2)	Extra personnel (1)
	Improved work organization (1)
No change (32)	
B. New work program^c^	
Negative impact (27)	Time-consuming (11)
	Additional work tasks (10)
	More stress (6)
Positive impact (9)	Improved work situation (4)
	Improved overview (3)
	Better understanding of patients’ situation (2)
Negative/positive impact (4)	Additional work tasks, but improved quality (4)
No comment on quality of impact (27)	

^a^1 missing (quantitative scale)

^b^Some HCWs listed several factors

^c^13 missing (quantitative scale)

For the new work program, about a third of the HCWs (24) perceived the new work program to have considerable impact on the work situation, while 20 and 23 HCWs perceived some or minor impact, respectively (13 missing). Table 2 (B) presents open comments concerning *how* the work program had an impact on the work situation (40 responses). The left column shows labelling of open comments as negative, positive or both negative/positive (27 HCWs, who primarily perceived the new work program to have minor impact on the work situation, did not comment on how the work situation was impacted). The right column shows the categorization of open comments related to each label, with the large majority of comments listing factors with perceived negative impact on the work situation (e.g., time-consuming, additional work tasks).

### Qualitative results

Table 3 summarizes interview results by showing the final template, differentiated by organizational level. Quotes illustrating inter-level discrepancies or similarities are representative for typical descriptions at each level. The final template was somewhat changed from the initial template by including an additional higher-order theme, resulting in three high-order themes; ‘strenuous work situation for HCWs’, ‘economy’ and ‘coping strategies’. The lower-order themes developed within each of them are presented next.

**Table 3:** Final coding template of interview data, differentiated by organizational level, with quotes to illustrate organizational discrepancies

Final template representing themes		Organizational level	Typical quotes illustrating inter-level discrepancies (examples for each level)
1. Strenuous work situation for HCWs			
1.1 Time pressure	HCWs:	Main stressor, Increasing	“It is the time pressure that wears us down, terribly. Yeah, it is tearing on us – it is the worst part, the absolute worst”.
	UL:	Main stressor, Increasing	“Some say there is more time pressure and stress now compared to how it used to be. That is probably correct, too”. “The time pressure is at the sacrifice of care. You calm and make people feel safe by holding their hand, so we (*HCS*) are probably heading in the wrong direction…”
	ML:	Improvement expected, cultural problem	“’We have never run so fast before!’ - this is a phrase, like many other expressions that are often incorrect”. “My experience is that a culture revolves around the focus on time pressure, a so-called ‘enjoying the misery’-culture”.
	ED:	No objective reason	“The HCWs complaining over more time pressure… It makes no sense. It is part of ‘the game’”.
1.2 Indirect task demands	HCWs:	Increasing amount	“Well, we have all these requirements directed at us, a lot more now than it used to be, about documentation and all kinds of stuff we must register and – we’re not able to do half of what we are supposed to…”; “It is at the expense of the patients, we need to take the time from somewhere, and then we steal it from them”.
	UL:	Increasing amount	“There is an increased demand of indirect tasks. It is very time-consuming, and we need more time (to accomplish these tasks)”. “We are required to spend 72 % of our workday on direct patient-related activities, but we can’t manage to do that. With all these indirect tasks, there is no chance”.
	ML:	Increased internal control	“There are no reasons for an increased need of time to indirect tasks (…) The requirements of documentation today are the same as previous years, but the internal control is stricter. The follow-up is tighter now. And will be getting even tighter”.
	ED:	Increased indirect time	“We see a registered increase in use of indirect time, but we don’t know whether this is due to an increase in indirect work tasks or as a consequence of improved registration”.
1.2.1 Work programs	HCWs:	New work tasks, time-consuming	‘More time-consuming, more documentation’. ‘A lot more responsibility and additional tasks’†
	UL:	Time and money-consuming	“’The program’†† was not supplied with any resources; it is eating up our time – tremendously. This is an example of a contradictory pressure between demands and results, in which we are decreed a lot of tasks, and not even a penny comes with it”. “This steals a lot of time and resources. Compulsory attendance here and there – this goes off our budget, but we have no choice”.
	ML:	Improved quality	“If they follow the checklists they will feel confident in doing their job. This is quality assurance”. “By doing it correct the first time they won’t have to do things twice”.
	ED:	(neutral)	“When they (*ML*) implement work programs, they must think that there is a gain to it. Maybe they (*HCWs*) think it is additional work”.
1.3 Organizational changes	HCWs:	Source of work strain	“There have been some organizational changes, you know, and it tears on us – When they start with all that, I’m just like: AGAIN!?”.
	UL:	Increased sick leave	“We had high sick leave for a while after the merger, and I definitely believe it was related to the merger and the subsequent effects; these things affect people; new routines, everything must be changed. It caused a lot of commotion, and it took some time before we were back on track; Even after 6 years it is still ‘them and us’, they are sitting on each side of the table”.
	ML:	Change is inevitable	“The nature of the home care services is to deal with changes and adjustments. That is home care”. “There are some myths out there; that organizational changes are terrifying. I believe it is crucial to adjust the organization”.
	ED:	N/A	N/A
2. Economy			
2.1 Budget model	HCWs:	Unfair model (time)	“I can drive 60 km and more during my evening shifts. It is NOT taken into account. Transferring is not taken into consideration on our work lists. We have protested against that many times, we think it is HIGHLY unfair!”
	UL:	Incorrect model (money)	“The budget model is not right. This district deals with a lot of psychiatry and since they are in good health physically, they score a very low ADL. They generate no money. It is wrong!”. “The real world is different from what the budget model tells us”. “A lot of the new work tasks are of indirect character not taken into consideration as they don’t generate any money”.
	ML:	Improved model	“We have been working very well for a long time on quality assurance and budget allocation based on patient weight; they get more resources if patient cases are heavy. We have a GOOD allocation of the resources available”.
	ED:	Misunderstandings on lower levels	“I can imagine this being a matter of discussion between the HCW and the unit leader. It seems to me that there are some misunderstandings of the model out there…”
2.2 Budget allocation	HCWs:	Poor economy, less money allocated	“You are told that the budget situation is getting worse and worse. And you are told that you have to do more and more in less time. It affects you, you feel; Ok, fine, there is a limit for – yeah – for what you can handle”. “Will there be directed even more requirements on us? I cannot run any faster and do my job any faster just because the council is short on money. It will make me ill, so I cannot do that”.
	UL:	Tight budget impossible to comply	“We notice a tightening, and we are under more supervision now. Now we have to hand over monthly reports about the financial condition”. “It is an invariable requirement that we stick with our budget. But we haven’t managed that. That is serious business, oh my, I think it’s horrible”.
	ML:	Increased allocation	“I believe many employees would say things have gotten worse, but that is just nonsense if you look at the budget increments”. “The budget allocations up till now have actually increased. We have objective figures that tell us that”.
	ED:	N/A	N/A
3. Coping strategies	HCWs:	Stress-reducing and time-managing	“We steal time from the patients (…) Or else we wouldn’t make it”.
	UL:	Reducing expenses	“My main goal has been to reduce excess spending”.
	ML:	Improving quality	“There is an increase in patient load. Patients return home sooner, in a worse condition. I believe that if they (*HCWs*) focus on following the checklists in the work program ††, they are safe”.
	ED:	N/A	N/A

*HCWs* Home Care Workers; *UL* Unit Leaders; *ML* Municipal Level; *ED* Economy Department

†Open survey responses, †† Anonymity

### Strenuous work situation for HCWs

*Time pressure* emerged as a main theme in the interviews, across all organizational levels. All HCWs spontaneously described their work day as busy and stressful, characterized by a constant fight against time. Several HCWs pointed at a negative trend towards increasingly hectic work situation. Increased demands of efficiency were perceived to be prioritized at the expense of compassionate care, which had to be downgraded due to lack of time. These descriptions were in accordance with accounts by unit leaders who in unison referred to employees expressing a feeling of being in a constant hurry and having too much to do. However, a few unit leaders qualified their accounts by pointing at behavior indicating a work situation not so stressful after all (e.g. HCWs coming in for lunch on time), claiming that a ‘stop watch-principle’ is a natural part of the job, and that the perception of time pressure is ‘highly subjective’. All unit leaders’ descriptions pointed at the same trend as the HCWs of increasing time pressure, and that work tasks were now more directed towards necessary medical treatment than traditional ‘caring’ activities. However, some descriptions were characterized by a more pragmatic flavor pointing at the necessity of change; indicating a more productive work organization whereby only medical, *documented* services were to be carried out (“*We are not hired to be social companions*”). Representatives at municipal level extended on the notion of time pressure being a subjective matter by pointing at a perceived destructive organizational culture worshiping negativity. HCWs’ complaints about lack of time were described as merely ‘phrases’ uttered with no basis in reality. Accordingly, all representatives at municipal level expressed doubt of an objective increase in time pressure for the HCWs, as “*no reasons for such an increase exist*”. Both work environment interventions and objective figures were referred to as reasons to expect the contrary. This was further supported by a representative from the economy department who also expressed a lack of understanding of HCWs’ perceptions of increased time pressure.

*Increased indirect task demands* emerged as a theme as some HCWs described a tendency towards a more challenging work situation characterized by increased demands of indirect time activities (i.e. documentation, writing reports, and transferring over a large geographical area). These activities were described as increasing sources of time pressure and stealing time from other (direct) work tasks, considered to be more important. Similar descriptions were presented by the unit leaders claiming that the services had become more comprehensive, involving additional areas of responsibility. At municipal level, however, descriptions regarding (direct and) indirect time were somewhat different, focusing more on developments in inspection plans and the controlling body as opposed to actual changes in work duties or workload. Documentation requirements were described as being the same as in previous years, yet with a more strict internal control, to achieve improved quality and thereby a positive change to the HCS. The representative from the economy department described a registered increase in use of indirect time, but could not say whether this was due to an increase in indirect work tasks (as stated by the HCWs) or improved registration (as required by the municipal level).

For the HCWs, the *new work program* was an example of a requirement directed from municipal level that entailed additional duties (indirect work tasks), resulting in increased workload and time pressure. Unit leaders, being responsible for managing the work organization and budget, expressed frustration regarding the introduction of the work program without supplementary resources. One unit leader illustrated this by referring to a consequent reduction in direct time from 63 to 49 % during implementation, implying that the new indirect tasks steal time from direct patient-related activities that constitute the basis for money allocation. One unit had to discontinue the implementation of the work program in order to secure “*the minimum requirement of producing services*” because of the additional demands placed on the HCWs. Another unit leader, who had assisted in composing the checklists, admitted that the program involved new, time-consuming work tasks, but she also pointed at benefits such as improved quality. This description was in accordance with representatives at municipal level who focused on quality assurance when describing the work program. Although agreeing that the program could be perceived as involving additional indirect tasks with no financial allocation, they emphasized the anticipated positive outcomes of the program such as systematization of work tasks and improved order and efficiency. However, when responding to a question on how the HCWs were to incorporate these additional tasks in practical terms, one representative stated that the work lists should be “*squeezed together*” and made tighter with *“extra assignments per work list in order to relieve others to enable time for documentation*”, implying increased efficiency demands for the HCWs.

*Organizational changes*, in particular unit mergers, were described by HCWs in all units affected as causing a more strenuous work situation. Consequences such as culture clashes, unit sizes too large, chaos, larger geographical distances, new localities, establishing and mastering new roles, and new ways of cooperating were cited as stressful factors wearing on them and being sources of increased time pressure. Unit leaders also described undesirable consequences of mergers, mainly in terms of increased sick leave and impaired work environment, with prolonged effects. One unit leader pointed at a severe budget overrun due to poor supervision and thereby loss of control as an example of a negative consequence of organizational changes. In contrast, representatives at municipal level were more visionary in their descriptions of organizational changes, focusing on enhanced quality, financial motives and an inherent need for change in the HCS. Though they did express awareness of adverse consequences for the HCWs and unit leaders (“*… there are expenses related to the employees and financial management*”), these consequences were brushed aside by focusing on the necessity for change and development.

### Economy

Two lower-order themes related to economy emerged in the interviews; budget model and budget allocation. The theme *Budget model* emerged as informants at all levels presented descriptions concerning the rationale for allocating time to work assignments and money to the units. The HCWs expressed a lot of frustration concerning indirect tasks not being included in the allocation of time. Only direct tasks were specified on their work lists and time to be spent on documentation, transferring, reporting etc. was not included in the time estimates. This was perceived as highly unfair considering the growing amount of such tasks that had to be carried out at the expense of direct-patient time (as described in two preceding themes). The key informant, who has done informal research on HCWs’ time distribution during their workday, supported the HCWs’ perceptions. Most unit leaders’ descriptions of the budget model were in accordance with the HCWs’, but their main focus was on money rather than time. Money was also a concern for some unit leaders when they described how the budget model was based on a perceived incorrect coupling between ADL and financial disbursement. Some patient groups were perceived to demand a lot of resources despite low ADL score, and unit leaders managing units in geographical areas with poor socio-demographic status regarded this to be highly unfair. This perception was in great contrast to the descriptions presented at municipal level focusing on the improved and now well-functioning budget model. One representative confirmed unit leaders’ perceptions of unfairness, but referred to objective figures stating otherwise. This representative, as well as the economy representative, hinted at a lack of understanding of the budget model among HCWs and unit leaders as the reason for frustrations.

*Budget allocation* involves descriptions concerning resource allocation in the HCS. Informants at all levels described an increased focus on financial issues. The HCWs described poor economy and budgetary constraints as the antecedents of impaired working conditions due to restrictions to hiring temporary workers, vacant posts and forced mergers. This was perceived to cause increased workload and sick leave, and the situation was considered to get worse every year. All units had struggled with budget overrun, and unit leaders described frustrations concerning a tight budget with financial demands that were perceived impossible to comply with. In turn, overspending involved a reduction of 3 % in next year’s budget allocation, making this a vicious circle. Reasons for budget overruns were mainly related to hiring of temporary staff (“*Last year this unit spent € 1 mill on temporary staff. That is a sick amount of money!*”), but also on car repairs, and compulsory projects and work programs, initiated by the municipal level, that required time and resources off the budget. Some unit leaders, like the HCWs, described a trend towards tighter budgets. One unit leader described a stable budget allocation suffering from increased efficiency demands, and another one to stricter control of reporting directed from the municipal level. In contrast, all municipal representatives described an increase in budget allocations, and one representative expressed awareness of an inter-level discrepancy in this regard. However, a couple of representatives described stricter economic control directed at the units, and tightening actions such as a reduction in time to carry out administrative (indirect) tasks, due to a more challenging financial situation for the municipality.

### Coping strategies

Informants at all organizational levels described making use of strategies or actions to deal with a strenuous work situation and a poor financial condition. The HCWs mainly described strategies utilized to cope with a strenuous work situation, involving both stress-reducing strategies and actions employed in order to get through the work list on restricted time. Stress-reducing strategies varied among the HCWs. Some described ‘active’ strategies (“*I call for assistance when I need it*”), other described ‘experience-based’ strategies (“*I am right here, right now, I cannot worry about the next patient on the list*”), and strategies of a ‘distancing’ character (“*I do what I’m supposed to do without engaging myself too much or else I get drained*”). Also, ‘task-minimizing’ coping strategies such as downgrading certain work tasks, skipping lunch, and cutting down time on assignments were described as essential actions in order to get through the work list on time. This was supported by the key informant who described employees distributing time where it is most needed, by either transferring time from one patient to another in more need, or from direct-time to indirect-time activities. He referred to an internal report revealing that HCWs shorten their assignments with 20–25 % on average. Unit leaders, on the other hand, mainly described actions to avoid excess spending, such as removing a car, reducing use of temporary workers, and avoiding employment of new workers in vacant positions. A few unit leaders also mentioned approaches to alleviate worker stress by actions such as hiding extra assignments by adding these to the work lists before the HCWs come to work in the morning, thereby making them unaware of the additional tasks. Municipal representatives described actions mainly focusing on quality assurance and improvement of the services by initiating different work programs. For example, in response to a potential increase in worker strain due to a new national reform, one municipal representative stated that checklists introduced in the new work program would ensure that the HCWs felt safe in their professional role when facing increased challenges in their work.

## Discussion

The results of this study support the initial assumption by showing that employees at different organizational levels in the HCS express divergent appraisals of factors related to the working conditions of HCWs, including impact of organizational measures. Follow-up survey responses supported the interview descriptions of HCWs, pointing at a further increase in time pressure due to rationalization-related measures. Some of the survey responses pointed at quality enhancing consequences of the new work program, as described by municipal representatives, yet the majority of survey responses revealed adverse strenuous side effects. We posit that organizational discrepancies as identified in the present study serve as an important barrier to a sustainable, well-functioning HCS in general and to quality-enhancing changes to work procedures in particular.

**Fig. 1:**
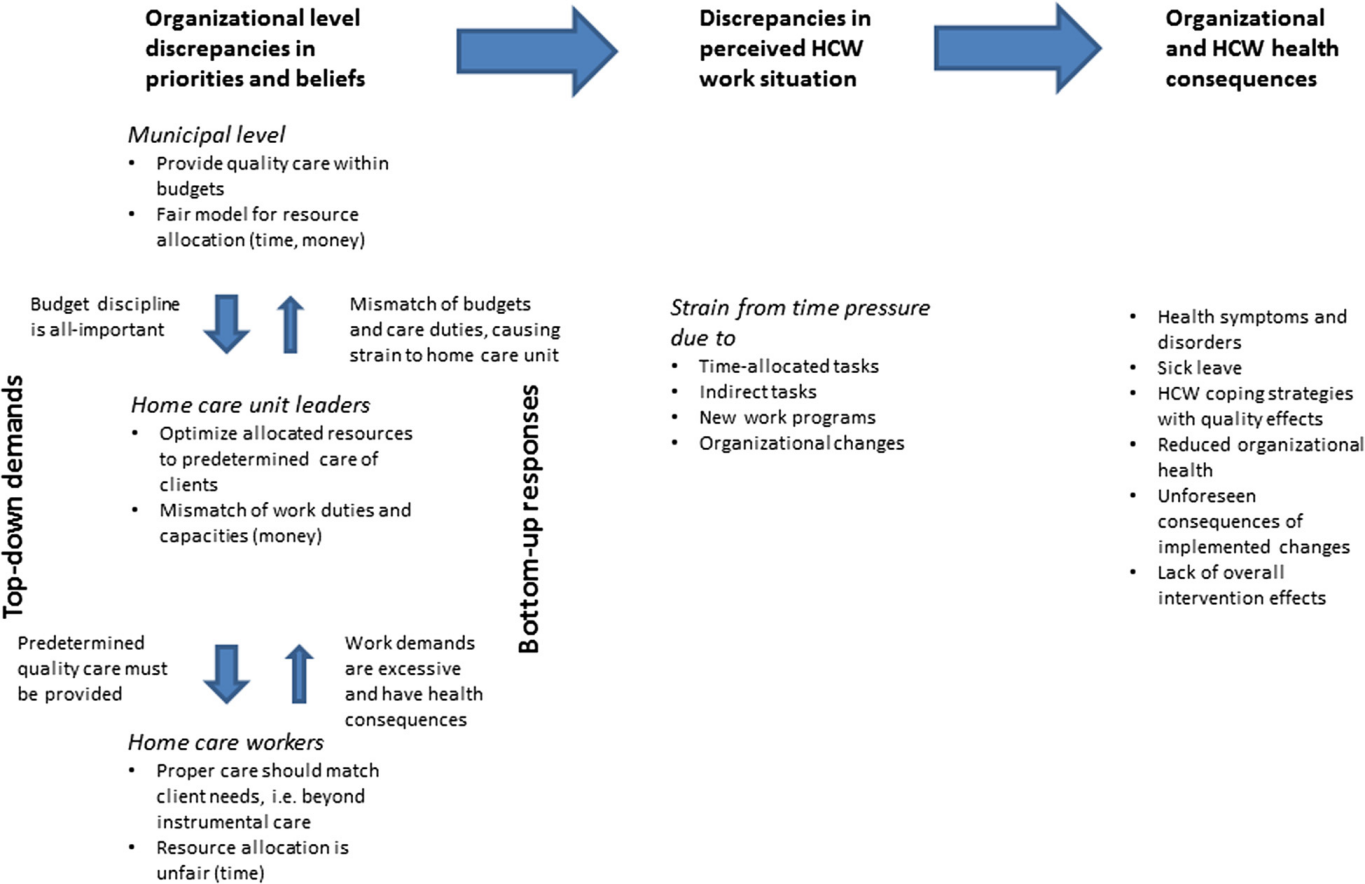
Graphic summary of results and inferences. Note: At each of the three organizational levels, upper and lower bullet points represent priorities and beliefs, respectively (left side). Primary communication across organizational levels is illustrated by vertical arrows. Bold arrows indicate dominant processes. Organizational discrepancies in priorities and beliefs result in further discrepancies in perceived work situation for HCWs (middle) and bring along adverse consequences for the organization and the individual worker (right side), treated in separate publications [6, 11]

Figure 1 presents a graphic summary of the study results and inferences: Organizational level discrepancies in priorities and beliefs concerning allocation of resources and the execution of care are identified. The top-down demands and bottom-up responses are incongruent, indicating a sub-optimal system involving stated demands exceeding perceived capacity. Primary communication across the organizational levels is illustrated by arrows, in which the top-down communication of demands is the strongest (demonstrated by bold arrows). The divergent appraisals of priorities and beliefs bring about further discrepancies in the perception of HCWs’ work situation, particularly with regards to strain from time pressure. Discrepancies in the appraisal of time-allocation, indirect tasks, and impact of a new work program and organizational changes translate into a straining work situation for the HCWs as the organization- and allocation-system is determined by decision-makers at a higher organizational level holding a divergent appraisal of significant matters. Finally, Fig. 1 illustrates how organizational discrepancy may result in poor organizational and employee health and functioning. In the following, the themes identified as areas of discrepancies will be discussed.

### Time pressure

A high level of time pressure has previously been identified in the HCS [4, 5, 20, 21], and particularly in the present case [11, 22]. Informants at all organizational levels agreed that time pressure was the most prevalent straining factor for the HCWs. However, the HCWs’ perceptions of their work situation as hectic and stressful were somewhat brushed aside by the management levels as being exaggerations and symptoms of an unfortunate culture, jargon and bad attitudes. These kinds of explanations may be held due to the considerable effort invested to alleviate precursors of time pressure, and hence improvement should be expected. Thus, HCWs’ perceptions of impaired working conditions are difficult to comprehend at municipal level for other reasons than being mainly a cultural problem. DeJoy [23] claims that the bias of ‘observers’ (i.e. managers; as opposed to employees being ‘actors’) toward internal attribution and underestimating external factors is likely to be exaggerated with increasing organizational distance. Accordingly, the municipal representatives elaborated on the interventions implemented (to argue for expected improvement) while the HCWs pointed at increased demands (to argue for perceived impairment). Unit leaders, being more closely connected to the daily work duties, supported the HCWs’ perception of increased time pressure and more ‘instrumental’ care. Although this was regarded as an unfortunate trend and a cause of distress for the HCWs, two unit leaders and all municipal representatives described this trend as necessary for improved quality of the Services in terms of being better organized and more efficient. The municipality changed the health division’s name from “Health and *Care* Services” to “Health and *Welfare* Services”, further signalling the downgrading of caring aspects. Erosion of the emotional aspects of home care work due to rationalization has been described in previous research (e.g. [8, 24]). The apparent organizational discrepancy regarding “quality of care” has been discussed as ‘instrumental’ versus ‘relational’ valued care provision [9, 25, 26] or ‘economical rationality’ (i.e. care based on resources available) versus ‘caring rationality’ (i.e. care based on patients’ needs) [27]. This discrepancy has implications for HCWs’ perceptions of quality on their job performance. Aronson and Neysmith [28] argue that the standardized, rational way of organizing home care work is difficult to translate into practice, and results in a discrepancy between HCWs’ official job descriptions and their own accounts of their work.

### Increased indirect task demands

Informants at all organizational levels agreed that there have been changes related to indirect tasks the last years. However, there is an organizational discrepancy concerning what this change involves in terms of added workload. HCWs and unit leaders described an increase in the amount of indirect tasks, resulting in additional time pressure for the HCWs. On the contrary, municipal representatives considered that the impact of changes was primarily stricter control by tighter follow-up of employees (more standardization in rationalization terms). However, this is likely to involve an actual increase in workload for the HCWs, as tighter control results in reduced flexibility; tasks are actually being carried out instead of postponed or given low priority due to time pressure. In this sense, increased control translates into increased requirements and workload for the HCWs, whereas the municipal level representatives can deny requiring additional work tasks, and thereby justify the decision of not allotting more resources. The lack of common understanding likely implies an added source of strain for HCWs. Worrall and Cooper [15] claim that top-level management, being removed from daily operations, are out of touch with the reality of the organization as perceived by the staff. It can be difficult to recognize practical implications in frontline work. Warren et al. [16] refer to striking organizational level differences in the recognition and reporting of occupational risk factors, in which employers seem to have a particularly difficult time identifying psychosocial risks.

### New work program

Different work programs have repeatedly been introduced in the Health and Welfare Services [11]. The results reveal organizational discrepancy in both focus and perceived impact of the new work program. The HCWs and the unit leaders presented similar descriptions, though the HCWs focused on increased workload and the unit leaders primarily focused on negative consequences related to work organization (in terms of releasing time to the increasing amount of indirect tasks) and budget. The municipal representatives acknowledged these changes, yet their focus was rather on quality assurance and increased efficiency. However, the responsibility of putting the work program into practice lies with the unit leaders, and the combination of increased workload, tighter work lists and no added financial resources makes it a tough priority for both unit leaders and HCWs. Due to stricter internal control of indirect tasks, the program results in increased strain for the HCWs and a source of frustration for the unit leaders. Decision-makers may be blinded by positive intentions and fail to realize the practical impact of such programs at frontline.

### Organizational changes

Similarly, a high degree of organizational discrepancy is identified in focus and perceived impact of organizational changes (e.g. unit mergers). The HCWs described how organizational changes directly and indirectly resulted in adverse consequences for their daily operations. Unit leaders focused more on subsequent consequences such as increased sick leave and budget overrun, as lie within their areas of responsibility and constitute objective indicators of their capabilities of managing the units. These consequences have been communicated to the municipal level, yet descriptions presented by the municipal representatives indicate that these reactions are not given much consideration. The discrepancies also indicate divergent perceptions of impact on service quality; while municipal representatives pointed to enhanced quality of the Services, the HCWs and unit leaders described negative consequences causing impaired quality within areas of their concern. This is in accordance with previous research showing organizational discrepancy in perceived impact of organizational change [15, 29, 30].

### Budget model

There is also a high degree of organizational discrepancy concerning the rationale for allocation of resources. The HCWs were most concerned with an unfair allocation of time, while the unit leaders focused on the allocation of money based on a perceived incorrect model. This is highly discrepant from the descriptions presented by municipal representatives. HCWs and unit leaders referred to “heavy” patient cases (e.g. psychiatric diagnoses, drug abuse) in terms of requiring time and money exceeding what is expected according to the ADL. Conversely, the municipal representatives referred to level of ADL as an indicator of functional level. As the budget model is based on the ADL, an organizational discrepancy in the understanding of what constitutes a “heavy” patient load may have impact on the appraisal of accuracy of the budget model.

### Budget allocation

Appraisals concerning budget allocation are to a large extent characterized by incompatible descriptions. The HCWs pointed at a reduction in budget allocation while municipal representatives claimed the opposite. Again, their descriptions are characterized by dissimilar focus, related to their areas of responsibilities: The HCWs focused on how poor economy had adversely affected their working conditions, the unit leaders expressed concern related to budget overrun, while municipal representatives referred to budget increases and optimistic objective figures. However, both unit leaders and municipal representatives described processes that might have resulted in an actual impaired economic situation for the units. For example, increased efficiency demands, several tightening actions and various compulsory work projects (e.g. projects on dental hygiene, protective footwear etc.) are concurrent requirements directed from the municipal level that may override resource increments. Accordingly, the increased focus on financial issues, restrictions to hiring temporary workers, stricter reporting control and budget overrun signalize an impaired economic situation to employees at lower organizational levels.

### Coping strategies

Organizational discrepancies are also identified concerning actions employed to cope with work-related challenges, such as types of strategies applied and for what purpose. HCWs focused on dealing with time pressure and a strenuous work situation, unit leaders on economic issues, and the municipal representatives on quality assurance. Again, this reflects their areas of responsibilities; the patients, the budget and the HCS, respectively. For the HCWs and unit leaders, it was the scarcity of resources (i.e. time and money) that needed to be managed in the most constructive way to be able to carry out their job satisfactorily; for HCWs sufficient time is a prerequisite in order to do ‘a good job’, for the unit leaders the ability to stick with their budget is an indication of doing ‘a good job’, while the municipality is responsible for ensuring ‘good quality’ of the Services. However, despite well-intended strategies of quality assurance (i.e. introducing work programs; stricter control), this has had adverse effects on HCWs and unit leaders by requiring more time and more money. Semmer [31] claim that negative intervention effects in terms of increase in time pressure and work load occur with some regularity.

### Suggestions for practitioners

The present results reveal several areas as possible targets for improvement in order to reduce organizational discrepancy and associated adverse consequences. Corrective measures should be introduced with an overall intention of developing intra-organizational ‘shared mental models’ of priorities and beliefs concerning matters of significance to the management of HCS (cf. Fig. 1). Improved communication channels across organizational levels likely involve increased sharing of knowledge and understanding of status quo at each level. This is particularly crucial for the HCWs’ responses as these seem under-communicated upwards in the system. The top level should be regularly connected to what is actually happening at frontline [15]. Warren et al. [16] suggest removing institutional barriers to the flow of knowledge in an organization characterized by discrepancy. In the present case, unit leaders are intermediaries and the strengthening of their position as messengers can facilitate the exchange of information across levels. In fact, the present HCS has arranged for weekly meetings of unit leaders and municipal representatives. However, New Public Management policies such as unit leaders being measured on budget figures and ‘unit performance’ may serve as obstacles to report on real unit conditions. Municipal representatives did express some awareness of HCWs’ perceptions, but didn’t seem to fully understand or acknowledge them as true. A more holistic view of how the organization is managed is recommended, especially during times of change, with increased focus on the relational, as opposed to technical aspects of management [15, 32]. Finally, the discrepancy between ‘objective job requirements’ and ‘personal job expectations’ likely involves emotional strain for HCWs [11]. Thus, clarification of *care* and *quality* is recommended to ensure common understanding and expectations with regard to work tasks and job performance (see also [27]).

These points are antidotes for this particular case, but should nevertheless be important to consider for organizations in general. Generalizability of the present results is limited due to the nature of case studies. However, general principles regarding the disruptive effect of organizational discrepancy to the management of organizations appear valid for most organizations and can probably be transferred to sectors outside health care and in other countries. The portrayals provided by the stakeholders at three organizational levels were elaborative and clearly discrepant though Norwegian municipalities (including the HCS) are characterized by a flat hierarchical structure [33, 34]. The disruptive effect of organizational discrepancy is likely even more significant in countries where organizations are characterized by greater organizational distance between decision-makers at top-level and workers at front line.

No considerable differences were detected amongst the units studied in this present study. Nevertheless, results of a previous study showed unit variations in effect of work environment interventions, in exposure to organizational changes, in sick leave rates and in excess spending and budget deficits. Other between-unit factors include geographical extension (impacting on transferring distances between patients’ homes) and patient characteristics (psychiatric diagnosis and drug addiction more densely populated in certain areas) [11]. Some of these elements have been treated in the qualitative themes ‘organizational changes’ and ‘budget model’. However, as the aim of the study was to explore the phenomenon of organizational discrepancy at three levels of an organization, the data was analyzed with regard to organizational levels irrespective of unit affiliation.

## Conclusions

The study shows that divergent priorities and beliefs of stakeholders at different organizational levels concerning factors significant to the management of the HCS and the performance of care bring about discrepancies in the appraisals of HCWs’ working conditions. Further, organizational discrepancy was identified to serve as an important barrier to a well-functioning organization and to quality-enhancing changes to work procedures. It is recommended to improve communication channels across organizational levels, and to facilitate the exchange of information regarding top-down messages (concerning ‘the system’) and bottom-up messages (concerning HCWs’ responses). Also, clarification of expectations to ensure a common understanding of care and quality is recommended. The results and inferences presented in this present study further suggest that prevalence and impact of organizational discrepancy should be included in future research on organizational issues.
